# Therapeutic Potential of Date Palm against Human Infertility: A Review

**DOI:** 10.3390/metabo11060408

**Published:** 2021-06-21

**Authors:** Maham Shehzad, Hina Rasheed, Summar A. Naqvi, Jameel M. Al-Khayri, Jose Manuel Lorenzo, Mohammed Abdulrazzaq Alaghbari, Muhammad Faisal Manzoor, Rana Muhammad Aadil

**Affiliations:** 1 National Institute of Food Science and Technology, University of Agriculture, Faisalabad 38000, Pakistan; mahamshehzad271@gmail.com (M.S.); hinarasheed345@gmail.com (H.R.); 2 Institute of Horticultural Sciences, University of Agriculture, Faisalabad 38000, Pakistan; 3 Department of Agricultural Biotechnology, College of Food and Agriculture Sciences, King Faisal University, Al-Ahsa 31982, Saudi Arabia; jkhayri@kfu.edu.sa; 4 Centro Tecnológico de la Carne de Galicia, Rúa Galicia Nº 4, Parque Tecnológico de Galicia, San Cibrao das Viñas, 32900 Ourense, Spain; 5 Área de Tecnología de los Alimentos, Facultad de Ciencias de Ourense, Universidad de Vigo, 32004 Ourense, Spain; 6 Department of Engineering, Applied Science University, 5055 Al Eker, Bahrain; mohammed.alaghbari@asu.edu.bh; 7 School of Food and Biological Engineering, Jiangsu University, Zhenjiang 212013, China; faisaluos26@gmail.com

**Keywords:** date palm, human infertility, natural remedies, fertility enhancers

## Abstract

Male and female infertility is a global major health problem. Approximately 15% of couples of a reproductive age are unable to achieve the desired pregnancy within 12 months, despite daily unprotected sexual intercourse, and about 10% of infertilities have no specific reason worldwide. Currently, many researchers are interested to investigate the use of natural remedies for preventive and curative purposes of infertility. This review brings together some of the data on the nutritional characteristics of the date palm and its different parts on fertility outcomes and critically evaluates the past and recent literature relevant to the consumption of date fruit against infertility-related problems. Due to its antioxidant potential, dates are considered a functional treatment for reducing the risks of infertility. In males, the date palm has a potent effect on the reproductive parameters including hormonal levels and seminal vesicle parameters as well as sperm motility, count, and viability; whereas, in females, it shows a convincing effect on reproductive parameters including oogenesis process, strengthening of oocytes, regulation of hormones, strengthening of pregnancy, reduction of the need for labor augmentation, and postpartum hemorrhage prevention.

## 1. Introduction

Infertility is defined as the inability of any couple having a pregnancy within 12 months of sexual intercourse after the use of protection measures [1]. It affects one in six couples and is recognized as a major health problem [2]. In developing countries, more than 186 million women suffer from infertility disorders [3]. Infertility affects millions of people all over the world. A considerable percentage of men who suffer from sexual dysfunctions experience harm to their subjective quality of life [4]. Furthermore, infertility rates are higher in other parts of the world, such as Eastern and Central Europe, Sub-Saharan Africa, Central, and South Asia, where they might exceed 30% [3]. Approximately, 15% of couples of a reproductive age are unable to achieve the desired pregnancy [5].

The term infertility and subfertility are used conversely. Infertility can be classified as primary and secondary. In primary infertility, a woman is not diagnosed with a clinical pregnancy and meets the criteria to be stated as infertile, whereas secondary infertility applies to the condition when a woman is diagnosed with a clinical pregnancy previously but is currently unable to establish a clinical pregnancy [6,7]. Primary infertility (57.5%) is much more common than secondary infertility (42.5%) [8]. Infertility is the major problem of human reproduction in different countries such as India, China, and Bangladesh. Different therapies are used to treat infertility. In assisted reproductive technology (ART) therapy, embryos are placed through a catheter in the uterine cavity. Women with ovarian endometriomas (cystic lesions that originate due to the disease process of endometriosis) achieve pregnancy through in vitro fertilization (IVF) treatment, which may not cause obstetrical complications [9]. Different therapies are used to treat infertility, but infertility has become an alarming problem that is prevalent in developing and developed countries. A study was conducted in 2010–2012 on 15,000 men and women in England, which stated that 12.5% of the women and 10.1% of the men experienced primary infertility [10]. In Pakistan, infertility prevalence is 22% with primary infertility comprising 4% of the total infertility cases [1]. 

**Figure 1:**
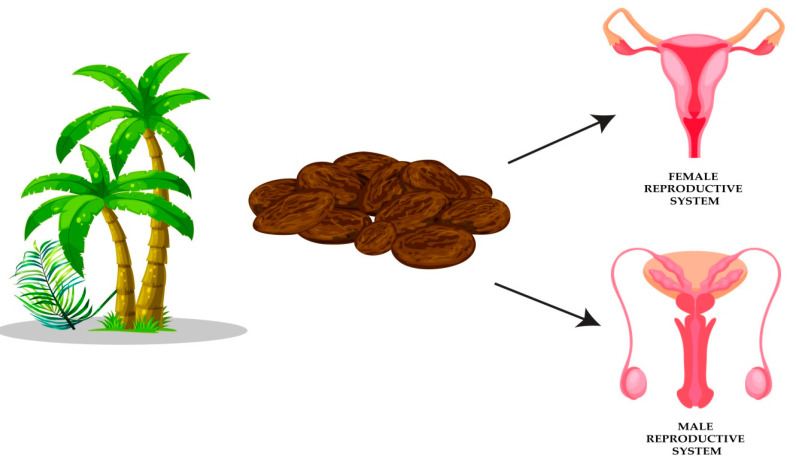
The graphical representation of the effect of date palm and its parts on the male and female reproductive system.

The extensive use of the date palm as a botanical and medicinal plant demonstrates its relevance to human health. Additionally, clinical trials have explained several benefits regarding date palm [11]. Pakistan is one of the major producers of date fruits in the world [12]. Dates are believed to be an important fruit crop globally, because of its excellent nutritional value and economic benefits. For the prevention and treatment of various health disorders, date palm (*Phoenix dactylifera* L.) parts, e.g., fruits, leaves, pits, and pollen, were used. These parts are the main source of available bioactive compounds, which are primarily responsible for their biological activities. Moreover, the date palm has been used throughout history to treat endocrine and reproductive system disorders [13]. The effect of date palm and its different constituents on the male and female reproductive system is shown in Figure 1. 

Date fruit has a pivotal role, possibly due to its high antioxidant activity, in facilitating male and female fertility [14]. This review highlights the improvement of fertility and other reproductive parameters in both genders. Date palm is an inexpensive, natural, accessible, and valuable food source, containing many bioactive components, which fight infertility. Therefore, this article presents the utilization of date palm to treat male and female infertility.

## 2. Causes of Infertility in Male

Globally, about 10% of infertility has no particular reason [15]. The Pakistani population belongs to the low-middle-income community, which has a high prevalence of infertility due to a lack of awareness and limited understanding of its causes, in addition to the lack of health-seeking behavior for this medical problem [1]. Various causes are attributed to the decline in sperm quality that may lead to infertility. Male infertility can be caused by defective spermatogenesis, ineffective transport, and ineffectual delivery of sperm. The presence of endocrine disorders such as hyperthyroidism and diabetes mellitus may cause azoospermia (failure to fertilize the ovum). Additionally, obstruction of the seminal vesicles and absence of the seminal ducts may affect the mobility of the sperm, causing infertility. Testicular disorders such as undescended testis might affect fertility [16]. During spermatogenesis, testicles produce sperm, and problems (chromosomal defects, trauma, infection, interaction with chemicals/radiation, varicocele, cryptorchidism) can cause infertility problems [17]. In conventional medicine, post-testicular causes of infertility including infection, injury, or seminiferous tubules obstruction, sperm motility, sympathetic nervous system damage, maturation disorders, and problems associated with the penis and physical disability also cause infertility in men [18]. Moreover, chronic and acute genital tract infections can also be a common cause of infertility in men [19]. Likewise, bacterial infections play a great part in the failure of the male reproductive system [5].

Physical issues can interfere with sperm development and obstruct the ejaculatory pathway. Varicocele is a condition characterized by sperm vessel enlargement, which is the most common cause of male infertility that affects around 40% of men [20,21]. Lifestyle factors that influence infertility include age (when starting a family), weight management, nutrition, and exercise. The male reproductive system is highly susceptible to environmental factors (cosmetics, pesticides, herbicides, preservatives, municipal and private wastes, cleaning materials, pharmaceuticals, and industrial by-products). These alien molecules enter our bodies in different forms that may lead to infertility [22]. Additionally, radiation exposure with a different type of radiation, such as electromagnet radiation, may cause ejaculatory disorders, reduce sperm production, and high doses lead to complete infertility [23].

## 3. Causes of Infertility in Female

According to the Center for Disease Control, female infertility can be caused by defective ovulation, transport (ovum and sperm), and implantation (zygote). Defective ovulation occurs due to the dysfunction of the hypothalamus and pituitary gland, which may prevent ovulation through the excessive production of prolactin [16]. Several known causes for female infertility are premature ovarian insufficiency, polycystic ovarian syndrome (PCOS), and endometriosis (a condition in which endometrial tissue grows on ovaries, the bowel, and the tissue lining of the pelvis) [24]. Around 40 to 50 % of women with endometriosis have an infertility problem [25]. Defective transport of ovum and sperm may also contribute to infertility and, as a result, the egg is trapped or not released, thus delaying conception. Abdominal surgeries cause scar tissue to develop that may alter the movement of the ovaries, fallopian tubes, and uterus. Like defective ovulation and transport, defective implantation may also lead to infertility through the congenital anomaly and fibroid formation. Defective implantation can occur due to congenital anomalies and fibroids near the cervix or fallopian tubes that may alter implantation of the zygote and cause infertility. Furthermore, fertilization may be hindered by psychosexual problems such as vaginismus or dyspareunia. Likewise, infection, surgery, trauma, and anti-sperm antibodies in the cervical mucus may also delay pregnancy [16].

Additionally, uterine flatulence is a form of complex dystemperament, which can trigger female infertility [26]. Poor lifestyle choices, such as eating patterns, stress, drinking, smoking, and obesity, can affect the general health and reproductive competence of a person and have been reported to significantly reduce the chances of pregnancy in women [27].

## 4. Date Palm: Nutritional Profile

The date is known for its rich source of macro-and micronutrients. It contains various minerals such as calcium (Ca), phosphorus (P), strontium (Sr), aluminum (Al), iron (Fe), potassium (K), copper (Cu), magnesium (Mg), manganese (Mn), and zinc (Zn) [28]. Natural antioxidants such as phenolic acids, flavonoids, and tannins, are abundant in dates [29]. Major sugar constituents (Glucose and fructose), amino acids (glutamine and aspartic acid) are present in various date cultivars. Moreover, large amounts of essential amino acids are present whereas histidine was also present in the lowest concentration [30]. Table 1 shows the nutrient profile of the date palm.

The male reproductive cells respond to date palm pollen (DPP), which is characterized by a high content of carbohydrate, lipid, and protein; hence, pollen should be highlighted as an ideal natural supplement containing energy and possessing good nutritional value [43,44]. The major constituents of date pollen are amino acids including glutamine, proline, glycine, leucine, tyrosine, phenylalanine, aspartic acid, threonine histidine, lysine, arginine, methionine, isoleucine, serine, alanine, and Valine [39]. Furthermore, pollen is a rich source of important vitamins, especially vitamin B (Thiamine, riboflavin, biotin, folic acid) and vitamin A [31,32,34]. The pollen also contains considerable levels of vitamins C and E. DPP also contains minerals such as Zn, selenium, Fe, molybdenum, Cu, Mn, cobalt, and fatty acids, including, linoleic, myristic, and palmitic acids [34]. In DPP extracts, significant amounts of rutin, flavonoids (lutein, naringin, isorhamnetin, and apigenin), and phenolic compounds (chlorogenic acid, catechin, gallic acid, quercetin, coumaric acid, caffeic acid) were identified [38].

On average, date leaves contained 4.8% crude protein, 31.9% crude fiber (neutral detergent fiber at 81.5%, acid detergent fiber at 59.8%, and lignin at 14.6%), and 12.9% ash (average Ca content of about 7 g/kg and P about 1 g/kg) on a dry weight basis [45]. Furthermore, date palm seeds constitute 10 to 15% of the weight of the date fruit depending on the variety [42]. Seeds had a low concentration of protein and lipid but contain dietary fiber and ash content. The seed has a very low sugar content, compared to the high sugar content in the date pulp. Date palm seeds are rich sources of many minerals, K content was highest, followed by sulfur, P, Ca, Mg, and sodium (descending order), while in trace minerals, the highest selenium content is followed by Fe, silicone, Zn, Cu, Mn, Sr, and Al in descending order [36]. Table 2 shows the proximal nutritional composition of different parts of the date palm. 

## 5. Date Palm Fruit, Pollen, and Seed: A Remedy against Infertility

**Figure 2:**
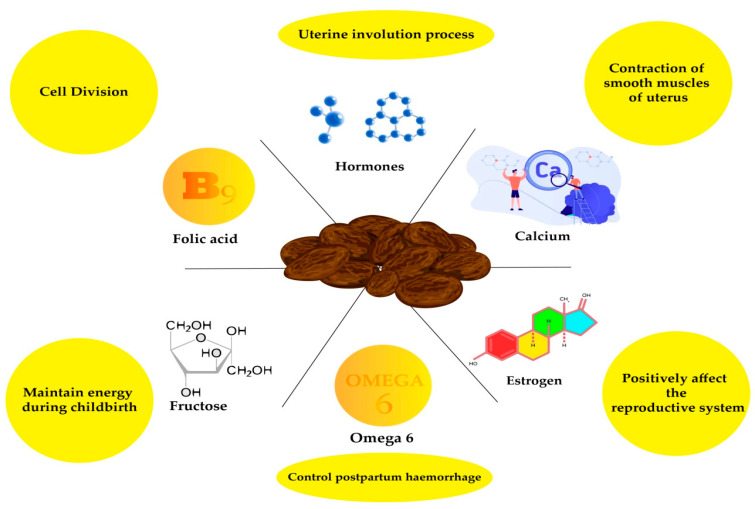
How date palm positively affects different parameters of fertility.

The date, an economical and natural food source, is an important nutritional fruit in various regions. Dates were used for both dietary and phytomedicinal purposes against several health disorders, such as infertility. Date fruit contains simple sugars that are a readily accessible energy source, strengthen uterine muscles, and provide energy to the mother during labor. It also contains hormones that help the uterus to stretch in preparation for delivery [48,49,50,51]. This section describes the health-promoting properties of bioactive components present in date palm attributed towards the betterment of fertility (Figure 2).

**Figure 3:**
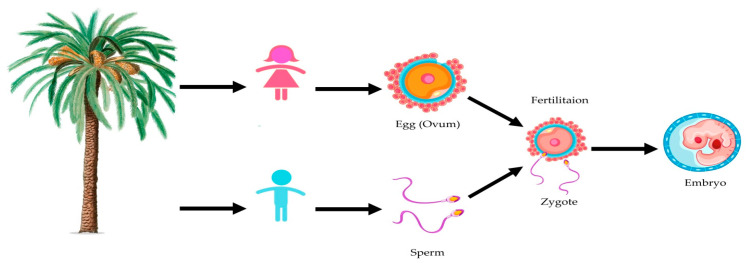
Illustration of the effect of date palm on sexual reproduction.

Moreover, early Egyptians and Chinese people used DPP as a rejuvenating medicinal agent and it is currently used as a dietary supplement globally [52]. DPP was traditionally believed to be an aphrodisiac and fertility enhancer and was recently identified as a traditional medicine for treating male infertility [53]. The aphrodisiac effect of DPP might be attributed to the presence of estrogen hormones. The pharmacological effects of DPP are not limited to males, as it has also demonstrated activities on gonadal stimulating potency and fertility promotion in women [54]. Date palm have a role in fertility improvement and sexual reproduction (Figure 3). Additionally, DPP can be used in the treatment of sexual incapacity and weakness in the Arab world and may cause a substantial rise in testosterone levels in oligoasthenozoospermic patients and follicle-stimulating hormone (FSH) levels in azoospermic patients [55].

Furthermore, frequent oral administration of date palm pits caused a substantial increase in the concentration of mean corpuscular hemoglobin, mean corpuscular hemoglobin concentration, and hemoglobin, whereas the total protein, alanine aminotransferase, and creatinine decreased significantly. Date seeds also have the potential to improve the testosterone level, serum biochemical values, and antioxidant status in testis [56]. Thus, date and other parts (DPP and seed) have wide therapeutic potential as a natural remedy against infertility in males and females. Table 3 shows the different bioactive components and mechanism of action of *Phoenix dactylifera* and its different parts.

### 5.1. Effect on Male Infertility

Various studies have been conducted that have shown the effect of the date palm parts on male fertility parameters. In a study conducted by Elberry et al. [71], an increase in the epididymis–body weight ratio or testis was observed due to the increase in the concentrations of estradiol and testosterone by DPP. Moreover, a study conducted by Mehraban et al. [72] designed a study to examine the effect of DPP and *Astragalus ovinus (A. ovinus)* extracts on fertility factors in male rats. *A. ovinus* extract was given at the dose of 500 mg/kg and DPP at the doses of 120 and 240 mg/kg. Their findings indicate that DPP improves fertility factors, whereas *A. ovinus* exhibited deleterious effects on sperm parameters and gonads in rats. In another study, the effect of *Eichhornia crassipes (EC)* (400 mg EC/kg body weight), *Saccharomyces cerevisiae (S. cerevisiae)* (120 mg/kg body weight), and date seed (0.2 mg/kg/body weight) were checked by using 40 male albino rats. The study results revealed that date seed significantly increased body weight gain. Moreover, date seeds and *S*. *cerevisiae* supplementation significantly increased the reproductive parameters [73]. Additionally, research on male adult rats was carried out to determine the effects of DPP on certain sexual behavioral parameters. Results showed that pollen increased the weight of testicles and serum testosterone levels of treated male rats. DPP may improve sexual activities in the cases of male infertility [69]. Table 4 represents the summary of the date and its different parts improving male fertility.

#### 5.1.1. Cadmium-Induced Infertile Male Rats

In a study conducted by Hassan et al. [80], testicular dysfunction-induced rats (*n* = 48) were studied. Oral administration of DPP results in rejuvenation of sperm counts, motility, sex organs weight, and testosterone level that were reduced by inducing cadmium chloride (CdCl_2_). DPP treatment also restored the reduced glutathione, catalase (CAT), and superoxide dismutase (SOD). In another study, the therapeutic potential of DPP was checked against cadmium (Cd) effects on adult male Wistar rats (*n* = 32). DPP treatment improved the reproductive damage and destructive effects of Cd on oxidative stress, spermatogenesis, and testis [65].

El-Komy et al. [78] carried out a study to determine the effect of DPP and seed extract of date palm on Cd-induced male albino rats (*n* = 36). Remarkable improvement was observed in luteinizing hormone (LH), aromatase enzyme, follicle-stimulating hormone (FSH), sperm quality, testosterone, estradiol (E2), total antioxidant capacity (TAC), xanthine oxidase (XO), glutathione, malondialdehyde (MDA), CAT, and SOD. DPP or date palm seed reduced the risk of Cd-linked infertility. Similarly, Hassan et al. [80] reported that DPP caused a significant enhancement in estradiol levels and sperm parameters in addition to a remarkable protective effect against testicular dysfunction induced by cadmium chloride.

#### 5.1.2. Streptozotocin-Induced Infertile Male Rats

El-Tahan et al. [81] performed a study on rats, which recommended that the intake of 5 and 10% of DPP extracts might be beneficial for diabetic-induced male infertility. Moreover, Kazeminia et al. [82] carried out a study to explain the effect of DPP extract in streptozotocin-induced male diabetic rats (*n* = 30) and found that DPP improved FSH LH and testosterone levels and may protect the testis structure. In another study, the effect of date pit powder was observed on the rejuvenation of reproductive functions in male diabetic rats. Their findings proved that the supplementation of date pit powder brings out a remarkable improvement in body weight, glycemic state, lipid profile, serum testosterone level, SOD activity, and sperm characteristics [79].

Another study was aimed to assess the effects of bee pollen (BP) and DPP suspensions (100 mg/kg body weight/day for 4 weeks) on streptozotocin-induced diabetic male Wistar rats. The results implicated that BP and DPP showed an antihyperglycemic effect through the normalization of testicular histological destructive changes, pituitary testicular axis dysfunction, and also showed improvement in the anti-oxidant system [77].

#### 5.1.3. Thyroid Disorder Induces Infertile Male Rats

Mehraban et al. [72] performed a study to examine the effect of DPP (ethanolic extract) on thyroid-induced male infertile rats that resulted in the restoration of genital sex organs weight, serum LH, FSH, T, sperm count, and motility due to induced hyper- and hypothyroidism. Another study was aimed to investigate the effect of DPP extract on the testicular dysfunction induced by a thyroid disorder. The results revealed that DPP extract may be played a protective role against testicular dysfunction [83]. Likewise, a study was carried out by El-Kashlan et al. [83] to examine the effect of DPP extract on testicular dysfunction of thyroid-disorder-induced male infertile rats. The result showed that the treatment with the extract of DPP prevented abnormalities induced through levothyroxine (LT4) or propylthiouracil (PTU). Supplementation of DPP extract to normal rats augmented serum levels of LH, sperm count, and motility as well as testicular antioxidant status. Additionally, it was proved that DPP extract caused a remarkable improvement in hyper- or hypothyroidism-induced sex hormones, testicular dysfunction, testicular marker enzyme activities, and sperm qualities [84].

### 5.2. Effect of Date Palm on Female Fertility Parameters

It is worth highlighting that date palm and its constituents have a significant effect on female fertility parameters. Moshfegh et al. [85] conducted a study to examine the impact of DPP on fertility and growth of the female reproductive system on Balb/c mice (albino, immunodeficient inbred strain). Results suggested that the use of DPP suspension during gestation and lactation substantially improves oogenesis. A study was conducted by Ali et al. [86] to check the effect of lyophilized extract (500 mg/kg) of date pits and plasma estradiol concentration (2 mg/kg) on the uterine rate of female rats. The result showed that the extract of the lyophilized date pit did not significantly affect the body or uterine weights. The polar fraction of the lyophilized extract greatly decreased the concentration of plasma estradiol. In contrast, the hormonal level was remarkably increased by the administration of non-polar fraction, but it was non-significant. Furthermore, pollen extracts showed significant improvement in antral follicles and other sex hormones in female Balb/c mice [87].

#### 5.2.1. Gestation, Labor, and Delivery

Abdelsalam et al. [88] conducted a study to examine the effect of probiotic fermented milk, Sukkary date fruit extract, and their mixture on hematological parameters of mature late pregnant Najdi ewes and Neonatal traits (*n* = 20). It was concluded that mean litter weight was significantly increased in ewes given dates alone; however, fermented milk or a mixture with dates did not significantly increase the litter weight although a slight increase was observed in ewes given a mixture. A study was carried out by Ahmed et al. [89] to check the effect of date fruit on the onset and progression of pregnant women’s labor (*n* = 89). The study result showed a positive impact on maternal outcomes (both the first and third stages of labor) and as well as fetal health factors. However, there was no significant difference observed regarding cervical dilation, maternal progression, and uterine contraction factors. Another study was performed to explain the effect of onset and augmentation of labor in nulliparous women (*n* = 154). Findings showed that date consumption reduces the need for labor augmentation but does not expedite the onset of labor [90]. Al-Kuran et al. [50] conducted a study to show the effect of date fruit on female labor parameters and delivery outcomes (*n* = 69). In the date fruit group, cervical dilation was also significantly increased, the requirement of oxytocin/protein was 28%, there was a higher rate of spontaneous labor, and the latent phase of the first stage of labor was shorter, as compared to the non-date fruit group. 

Moreover, another study was carried out to identify the effect of eating dates and drinking water during labor versus Intravenous (IV) fluids on labor and neonatal outcomes. The results of the study showed a significantly shorter median duration of the second and third stages of labor among the study group compared to the control group with no significant harm regarding the mode of delivery and neonatal outcomes [91]. Besides, Kordi et al. [92] performed a study to check the effect of date fruit on the duration of labor in Nulliparous women (18–35 years), who were in their 37–38th week of pregnancy. The result showed no significant difference between the average lengths of the active phase of labor in the two groups. Taavoni et al. [93] carried out a study to determine the effect of date palm syrup on labor pain in nulliparous women (*n* = 80). This study outcome showed that date palm syrup significantly lowered labor pain and starting impact occurred late by the usage of palm syrup, but it has a long relief effect.

#### 5.2.2. Pre-Eclamptic Pregnant Women

Royani et al. [94] conducted a study to evaluate the potential effect of Ajwa dates on the prevention of preeclampsia threats in pregnant women. Findings have shown that the daily intake of 7 Ajwa dates has a remarkable impact to decrease the Roll-over-Test (ROT) and Mean arterial pressure (MAP) in pregnant women at the risk of developing preeclampsia, thereby helping to prevent preeclampsia from developing.

#### 5.2.3. Prevention of Postpartum Hemorrhage

Another aspect attributed to date palm is the prevention of postpartum hemorrhage [95] carried out a study that aimed to determine the effect of date fruit in nulliparous women (*n* = 100) on the amount and duration of postpartum bleeding. The results indicated that the date fruit was not effective in reducing the number of bleeding days. However, it reduced the amount of bleeding. Another study was designed to elucidate the effectiveness of dates on postpartum hemorrhage (*n* = 34). Findings clearly highlighted that date was more effective than the infusion of oxytocin on postpartum hemorrhage [96].

Khadem et al. [58] conducted a study to check the effect of date fruit on a postpartum hemorrhage. Results manifested that the mean of blood loss in the date group was significantly less than the oxytocin group in the entire three hours after delivery. Another study was designed by [61] to examine the effect of the date on postpartum hemorrhage (*n* = 60). From 37 weeks of gestation to delivery, the treatment group was given 7–9 dates/day. Findings showed that there were no major variations in estimating blood loss in the type of delivery and course of labor. In the treatment population, an effect on the period of labor was observed.

#### 5.2.4. Experimentation with Infertile Adult Female Rats

Hammed et al. [97] conducted a study to determine the effect of DPP suspension on adult female rats exposed to lead acetate. Results showed that oral administration of a protective dose of DPP suspension (150 mg/kg body weight) could lead to a re-balancing of the harmful effects. Another study was carried out to compare the effect of ethanol extract of date palm fruit and propolis on fertility in female mice. Khalal date fruit (ethanolic extract) and propolis can increase the number of ovarian follicles. It was observed that ethanol extract of Khalal date fruit dose can increase the number of ovarian follicles higher than propolis [98].

A study was conducted to investigate the effect of date fruit on the reproductive process of female rats. Results showed that there was a significant improvement in hormonal regulation, strengthening of oocytes, and pregnancy [99]. Moreover, concomitant supplementation of barley and date fruit to the hypercholesterolemic mice group revealed remarkable improvement in ovarian function and structure [100]. Table 5 shows the summary of date palm and its constituents improving female fertility parameters.

## 6. Conclusions

It can be concluded that date fruit, its various parts (DPP, date pit, leaf), and extract contain many beneficial components that play a vital role in fertility. This review mainly focuses on information regarding dates and their favorable impact on male and female fertility as it is an effective nutritious plant that is easily accessible. Dates are a valuable source of macronutrients (simple sugar, amino acids, and fatty acids), phytochemicals (polyphenolic components, flavonoids), carotenoids, saponins, tannins, vitamins, and minerals that have a significant role in the body. Due to its wide-ranging therapeutic ability, date palm may contribute towards the betterment of fertility. In males, it exhibits a positive impact on hormonal levels, seminal vesicle parameters, sperm motility, and its count and viability. Likewise, in female, dates have a convincing effect on reproductive parameters (oogenesis process, strengthening of oocytes, regulation of hormones, strengthening of pregnancy). This review also discusses that dates may reduce the need for labor augmentation and prevents postpartum hemorrhage. Many researchers are currently trying to evaluate the efficacy of natural remedies for both preventive and treatment purposes of infertility. Although the beneficial effect of dates and their products has been studied, most of the previous research studies were conducted on rats. Furthermore, when querying various databases, it was found that there are limited human studies, so further studies on humans are required to identify the effective form of the date and its suitable daily intake to prevent and treat infertility in humans and more study on female infertility, especially associated with pre-eclampsia, is also needed.

## Figures and Tables

**Table 1:** The nutrient profile of the date palm.

Main Group	Nutritional Profile	Reference
Vitamins	Vit A, Vit E, Vit C, Vit B1, B2, B3, B6, B7, B9, Carotenoids (such as lutein, β-cryptoxanthin, and β-carotene)	[31,32,33,34]
Minerals	Al, Ca, Cu, Fe, K, Mg, Mn, P, Sr and Zn, Se, Mb, Co, Si	[34,35,36]
Phytochemicals	Flavonoids (isorhamnetin, apigenin, lutein, and naringin), and phenolic compounds (caffeic acid, gallic acid, catechin, coumaric acid, chlorogenic acid, and quercetin), tannins and anthocyanins, rutin	[37,38]
Protein	Amino acids (aspartic acid, threonine, glutamine, proline, glycine, alanine, Valine, methionine, isoleucine, leucine, tyrosine, phenylalanine, Histidine, lysine, arginine, and serine)	[39]
Fatty acid	Unsaturated fatty acids (oleic, linoleic, and Linolenic acids) Saturated fatty acids (palmitic, linoleic, myristic acids)	[34,40,41]
Carbohydrates	Soluble sugars, dietary fiber	[42]

Aluminium: Al; Calcium: Ca; Copper: Cu; Iron: Fe; Potassium: K; Mmagnesium: Mg; Manganese: Mn; Phosphorus: P; Strontium: Sr; Zinc: Zn; Selenium: Se; Molybdenum: Mb; Cobalt: Co; Silicon: Si.

**Table 2:** The proximal nutritional composition of different parts of date palm.

Date Palm Parts	Nutritional Composition	References
Palm pollen grains	Moisture (28.80%), ash (4.57%), crude fiber (1.37%), crude fat (20.74%), protein (31.11%) and carbohydrate (13.41%)	[46]
Date seed	Moisture (3.1–7.1%), protein (2.3–6.4%), fat(5.0–13.2), ash(0.9–1.8%) and dietary fiber (22.5–80.2%), phenolics (3102– 4430 mg gallic acid equivalents/100 g), antioxidants and dietary fiber (78–80 g/100 g)	[47]
Date leaves	Crude protein 4.8%, crude fiber 31.9% (Neutral detergent fiber 81.5%, Acid detergent fiber 59.8%, Lignin 14.6%), ash 12.9% (average Ca content about 7 g/kg and P about 1 g/kg) on a dry weight basis	[45]

Calcium: Ca, phosphorus: P.

**Table 3:** The bioactive components of date palm and their mechanism.

Date and Date Parts	Active Component	Mechanism	Reference
Date	Simple sugar	Dominant and readily accessible source of energy that provides, save and maintain the mother’s power during labour	[49,50,51]
Date	Glucose	Provide energy, strengthens uterine muscles, and best nutritional material for cervical dilutions	[41,50,57]
Date	Ca	Contraction of the smooth muscle of the uterus	[58,59]
Date	Tannin	Contractions of smooth muscles of the cervix	[58,60]
Date	Tannin and Linoleic acid	Control bleeding	[57]
Date	Sugar, Vitamin B1, and Fe	Control the rate of movement of the uterus	[61]
Date	Folic acid	Role in cell division and the formation of the genetic structure of cells	[62]
Date	Potuchin hormone	Potuchin hormone serves to bind the uterus and muscles of the uterus so that it can help reduce postpartum bleeding	[61]
DPP	Estrogenic compound: estradiol, estriol, and estrone	Alleviate infertility through their gonadotrophic activity in male rats	[63,64,65]
DPP	Estrogen compounds	Estrogen compounds increase the estrogen hormone. These compounds transfer to embryos and offspring via lactate and placenta and affect the reproductive system in adult mice	[66]
DPP	Saponins	Saponins encourage the Leydig cells of the testes to increase the testosterone production system	[67,68]
DPP	Carbohydrates, Saponins and gallic tannins	DPP has an aphrodisiac potential and may increase the reproductive parameters of male adult rats	[69]
DPP	Estradiol components	Play a role in regulating the renewal of spermatogenic cells and male reproductive tissues that possess oestrogen receptors	[50]
DPP grains	Phytochemicals: alkaloids, saponins, and flavonoids	Phytochemicals have engorgement and androgen enhancing properties that improve sexual behavior in male rats	[66]
DPP grains extracts	Estrogenic materials	Gonad stimulating compounds that improve male infertility	[70]

Date palm pollen: DPP; Calcium: Ca.

**Table 4:** The summary of date and its different parts improving male fertility.

Date and Date Products	Subjects	Target	Materials and Methods	Active Component	Result	References
*Phoenix dactylifera* (Date)	Wistar rats	On the prostate and seminal vesicle of Wistar rats	*n* = 20 Group 1 (control) Group 2 to 4 (experimental group) received the extract at 250, 500, and 1000 mg/kg body weight orogastrically for 35 days	Vitamins, Simple sugar, Flavonoids Saponins, Tannins, Carotenoid-s and steroids	The extract might affect sperm function by reducing sperm quality and viability	[74]
DPP	Pre-pubertal rats	Serum testosterone levels and body weight	Four groups (12 pups each) (Control 1, Experimental 1, Control 2, and Experimental 2). Experimental groups 1 and 2 were given oral DPP suspension of 120 mg/kg daily for 18 and 35 days respectively	−	Increase in serum testosterone levels with a concurrent increase in body weight	[75]
DPP	Infertile men	On male infertility	Pollen powder (500 mg capsules twice daily for 3 months)	−	The treatment significantly increased serum (LH), (FSH) and testosterone levels, sperm count, and motility. Sexual desire was also increased significantly increased wives of two treated males become pregnant during the treatment period.	[76]
DPP extract	Thyroid disorder induces male rate	Protective effect on testicular dysfunction	Six groups; Group 1 (Control) Group 2 (DPP extract, 150 mg/kg) Group 3 (hyperthyroid received intraperitoneal injection of L-T4, 300 μg kg^−1^) Group 4, received L-T4 plus DPP extract, Group 5 (hypothyroid received PTU 10 mg /kg) Group 6 received PTU plus DPP extract. Treatment was given for 56 days	−	Supplementation of DPP extract to normal rats augmented sperm count and motility, serum levels of LH, testosterone, and estradiol (E2) paralleled with increased activities of 3β-hydroxysteroid dehydrogenase and 17 β-hydroxysteroid dehydrogenase as well as testicular antioxidant status	[58]
Bee Pollen and DPP	Streptozotocin-inducing diabetic Wistar rats	Male sexual dysfunction	Bee pollen and DPP suspension (100 mg/kg body weight /day for 4 weeks	Antioxidant Compounds (Phytoestrogen and flavonoid)	BP/DPP suspension may have a protective role against diabetic induced pituitary testicular dysfunction and testicular histological changes in association with antihyperglycemic actions	[77]
DPP and seed extract	Cadmium-induced female rats	Check protective effect on cadmium induce infertility	Six groups (36 albino rats) G 1 (control) G 2 (DPP): (240 mg/kg) daily, G 3 (DPS):(100 mg/kg) daily, G 4 (CdCl_2_): (5 mg/kg) every other day Group 5 (CdCl_2_ + DPP): CDC12 as group 4 and DPS as group 2 Group 6 (CdCl_2_+dps): CdC12 as group 4 and DPS as group 3 Treatment period: 30 days	Antioxidant	There was a significant improvement in these parameters (sperm quality, T, E2, FSH, LH aromatase enzyme, TAC, GSH, SOD, CAT, MDA, XO testis histoarchitecture) in CdCl_2_ treated rats	[78]
Date palm seed extract	Male rats	Date palm seed extract effect on Hematological parameters hormone testosterone and antioxidant status in testis.	Twenty male rats (10 rats in each group) Group 1 (Control) Group 2 received date seed extract in a dose of 2 ml/kg orally for 60 days	−	The daily oral administration of seed extract decreased malondialdehyde level in testicular tissues. It has the potential to improve serum biochemical values, testosterone level and antioxidant status in testis.	[56]
DPP and *Astragalus ovinus*	Adult Male rats	Effect of DPP and (A.Ovinus) on fertility in healthy adult male rats	Thirty-six rats (six groups) One group (Control) Other five groups (Treatment) DPP (120, 240 and 360 mg/kg) and *A.* ovinus (100, 500 mg/kg) were given orally.	−	Findings indicate that DPP could improve fertility factors, while *Astragalus ovinus* can exhibit deleterious effects on gonad and sperm parameters in rats	[72]
Date pit powder	Male diabetic rats	Restoring reproductive function	Group 1 (control) Group 2 (diabetic rats) fed a basal diet, Group 3,4 and 5 fed basal diets supplemented with DP at three-levels (5, 10 and 15%)	Antioxidants	Supplement of DP causes a significant increase in body weight, remarkable improvement in sperm characteristic and glycemic state, an increase in serum testosterone level a decrease in Thiobarbituric Acid Reactive Substances (TBARS) and an increase in SOD activity in testicular tissue, and improvement in lipid profile	[79]
Dietary supplement (Date seed, *Saccharomyces cerevisiae,* and normal basal diet)	Male rats	Reproductive and productive performance	*n* = 40 male albino rats Groups (4) Control group, EC supplement group (400 mg EC/kg body weight) *Saccharomyces* *cerevisiae* supplement group (120 mg/kg body weight) Date seed supplement group (2.0 mg/kg body weight)	−	Date seeds supplementation significantly increased body weight. Date seeds and *Saccharomyces cerevisiae* supplements increase significantly gonadosomatic TAC and reproductive parameters.	[73]

Date palm pollen: DPP; luteinizing hormone: LH; follicle-stimulating hormone: FSH; levo thyroxine: L-T4; bee pollen: BP; Date palm seed: DPS; cadmium chloride: CdCl_2,_; estradiol: E2; T, testosterone; total antioxidant capacity: TAC; gonad stimulating hormone: GSH; superoxide dismutase: SOD; catalase: CAT; malondialdehyde: MDA; xanthine oxidase: XO; *Eichhornia crassipes*: EC.

**Table 5:** The summary of date palm and its constituents improving female fertility parameters.

Date and Date Products	Subjects	Target	Material and Method	Active Component	Result	References
Date fruit	154 Nulliparous women	The onset of labour and the need for induction	Date consumer (77) and Date control group (77) Intake (7 dates/day)	−	No significant difference in the onset of spontaneous labour. Date consumption reduces the need for labour augmentation but does not expedite the onset of labour	[90]
Date fruit	Females	Labour parameter and delivery outcomes	Date fruit group: 69 women for 4 weeks per day before labour Non-date fruit group: 45 Intake (6 dates/day for 4 weeks)	−	In the date fruit group, cervical dilation was significantly increased, there was a higher rate of spontaneous labour and the latent phase of the first stage of labour was shorter 28% of women in this group required the use of protein or oxytocin compared with 43% in the non-date fruit group	[50]
Date fruit	Nulliparous women (18–35 years) who were in their 37–38th week of pregnancy	Duration of labour	Control group Date consuming group (70–76 g dates daily for from the 37th week of pregnancy)	Tannins	There was no significant difference between the average length of the active phase of labour in the two groups	[92]
Date fruit	Pregnant women	Onset and progression of labour	89 participants Control group: (31 women) Date consuming group: 26 women (7 dates/day) Dates + water consuming group: 32 women (7 dates + 250 mL)	−	Significant positive impact on maternal outcomes on both the first and third stage of labour and fetal well-being factors No significant difference between the date fruit consumer and their counterparts regarding cervical dilation, the regularity of uterine contraction, and maternal progression factors	[64]
Date fruit	Pregnant women	Postpartum Haemorrhage	Group 1 (50 g oral deglet Noor dates Group 2 (10 units of Intramuscular oxytocin)	Serotonin	In the whole three hours after delivery, the blood loss means in date group was significantly less than the oxytocin group	[58]
Date fruit	Pregnant women	Bleeding, length of labour, type of labour	Total of 60 Treated Group (30) consumed 7–9 dates per day since 37th-week gestation Control group (30)	−	The result of the study about the length of labour showed that there was an effect of data consumption on the length of labour with a value of *p* = 0.000	[61]
Date fruit	Pregnant women	Preeclampsia	40 Pregnant women were randomly assigned to Control group (10) Intervention group (30) Intake (7 dates/day for a week)	−	Daily consumption of 7 Ajwa date Has a remarkable potential to decrease MAP and ROT in pregnant women at risk of developing preeclampsia and thus prevent from preeclampsia	[94]
DPP	Female reproductive study	Reproductive System	Intake (100 and 200 mg/kg)	Flavonoid, Alkaloid, and Estradiol	The use of DPP suspension during gestation and lactation increase oogenesis significantly	[85]
DPP	Adult female Albino rats exposed to lead acetate	Ovarian function and fertility	Total = 404 (4 groups) Control Group (orally 1 mL distilled water) T1 was given orally 150 mg/kg BW. DPP (0.5 mL) T2 was given orally 10 mg/kg BW. Lead acetate 1 mL T3 was given oral administration of both DPP 150 mg/kg BW and 10 mg/kg BW. lead acetate All animals were treated via gavages needles for 6 weeks	−	Oral administration of DPP with a protective dose of 150 mg/kg BW lead to rebalancing the harmful effect of lead acetate in female rats	[97]
Date palm syrup	Nulliparous Women	Labour pain	Total = 80 Control Group: Water Palm Syrup Group: Consumed pulp-free syrup added in 150 mL water	−	Date palm syrup significantly reduces labour pain. Findings showed that starting impact occurred late in palm syrup usage, but it has a long relief effect	[93]
Probiotic fermented milk, Sukkary date fruit extract, and their mixture	Mature late pregnant Najdi ewes	Neonatal traits Hematological Parameters	Total = 20 Group 1 (control) Group 2 (50 mL date extract every other day for the last 8th week of pregnancy Group 3 (50 mL of probiotic fermented cow’s milk for the same period Group 4 (50 mL of the mixture Date extract: Fermented milk, 1:1)	−	Mean litter weight increased significantly in ewes given dates alone (85% more kg than control) Fermented milk or mixture with dates did not significantly increase the litter weight. Fermented milk alone did not show alteration in litter birth weight	[88]
Barley and date fruit (Anti hypercholesteremia Impact)	Female Wistar Albino Rats	Ovarian function and infertility	Eight Groups (*n* = 12) Control (C) Barley Group (B): Diet containing 10% barley grains Date Palm fruit group (D): A diet containing 10% fruit Barley and date group (BD) High cholesterol diet group (H) High cholesterol and barley grains (HB) High cholesterol and date palm fruit group (HD) High cholesterol and both barley and date palm fruit (HBD)	Phytomicro nutrients polyphenols, Beta-glucan, and trace elements	Concomitant supplementation of barley and date fruit to the hypercholesterolemic group revealed marked improvement of ovarian structure and function	[77]

DPP, date palm pollen; mg, milligram; kg, kilogram; BW, body weight; ROT, Roll-over-Test; MAP, mean arterial pressure.
